# *Piscine orthoreovirus* (PRV) in red and melanised foci in white muscle of Atlantic salmon (*Salmo salar*)

**DOI:** 10.1186/s13567-015-0244-6

**Published:** 2015-09-08

**Authors:** Håvard Bjørgen, Øystein Wessel, Per Gunnar Fjelldal, Tom Hansen, Harald Sveier, Håkon Rydland Sæbø, Katrine Bones Enger, Eirik Monsen, Agnar Kvellestad, Espen Rimstad, Erling Olaf Koppang

**Affiliations:** Institute of Basic Science and Aquatic Medicine, School of Veterinary Medicine, Norwegian University of Life Sciences, Oslo, Norway; Institute of Food Safety and Infection Biology, School of Veterinary Medicine, Norwegian University of Life Sciences, Oslo, Norway; Matre Research Station, Institute of Marine Research, Matre, Norway; Lerøy Seafood Group ASA, Bergen, Norway; Department Brandasund and Rex Star, Lerøy Sjøtroll AS, Skjervøy, Norway; Lerøy Aurora AS, Skjervøy, Norway; Lerøy Aurora AS, Tromsø, Norway

## Abstract

Melanised focal changes (black spots) are common findings in the white skeletal muscle of seawater-farmed Atlantic salmon (*Salmo salar*). Fillets with melanised focal changes are considered as lower quality and cause large economic losses. It has been suggested that red focal changes (red spots) precede the melanised focal changes. In the present work, we examined different populations of captive and wild salmon for the occurrence of both types of changes, which were investigated for the presence of different viruses by immunohistochemistry and RT-qPCR. The occurrence of red or melanised foci varied significantly between the populations, from none in wild fish control group, low prevalence of small foci in fish kept in in-house tanks, to high prevalence of large foci in farm-raised salmon. Large amounts of *Piscine orthoreovirus* (PRV) antigen were detected in all foci. No other viruses were detected. Red focal changes contained significantly higher levels of PRV RNA than apparently non-affected areas in white muscle of the same individuals. Some changes displayed a transient form between a red and melanised pathotype, indicating a progression from an acute to a chronic manifestation. We conclude that PRV is associated with the focal pathological changes in the white muscle of farmed Atlantic salmon and is a premise for the development of focal melanised changes.

## Introduction

Farmed Atlantic salmon may develop melanised focal changes in the white muscle [1]. The condition represents a considerable challenge for the industry with as much as 20% of the fillets at Norwegian processing plants reported to be affected [2]. Changes typically appear as distinct areas within the white muscle, frequently located in the cranioventral and craniodorsal regions of the abdominal wall, but may also be found elsewhere in the musculature [1,3]. This condition causes large economic losses as fillets with pigmentation disorders are downgraded. Melanised focal changes are common in farmed Atlantic salmon all along the Norwegian coast. However, the prevalence is negligible in farmed rainbow trout (*Oncorhynchus mykiss*) although they are produced under similar conditions (H. R. Sæbø, Lerøy, personal observation). Additionally, there are no reports of melanised focal changes in wild-caught salmon (E. Sterud, Norwegian Salmon Rivers, personal communication). Melanised muscle changes were reported in a study addressing the tagging of wild salmon [4], but with a visible physical foreign body present, this condition is not comparable with melanised focal changes as described previously [1].

The discolouration is attributed to melanin-producing leukocytes referred to as melano-macrophages [5-7]. Normally, such cells are prevalent in lymphoid organs of fishes [6,7], but accumulation of melano-macrophages may be part of chronic inflammatory responses such as peritonitis induced by oil-adjuvanted vaccines [8]. Macrophages use oxidation to inactivate pathogens, and there is a particular need for protection against oxidative damage in chronic inflammatory processes. In contrast to mammals, populations of leukocytes in ectothermic vertebrates produce melanin, which is a powerful anti-oxidant. It has been proposed that these melanin-synthesising mesenchymal-derived cells could be classified as melanocytes [9].

The aetiology of the focal melanisation in the white muscle has remained obscure. It was previously suggested that the changes might occur due to chronic inflammatory processes induced by dislocation of oil-adjuvanted vaccine components [1]. Nevertheless, later studies showed that also un-vaccinated fish may develop similar lesions at the same prevalence, and the hypothesis of a vaccine-induced aetiology was abandoned [10-12]. Other explanations that have been suggested point towards mechanical trauma due to handling of the fish, causing focal intramuscular haemorrhages that later melanise. However, these assumptions have not been documented by histological investigations. Besides, intramuscular haemorrhages and haematoma do not initiate granulomatous inflammation.

Melanised, but not red, focal changes have been investigated by different morphological and transcriptional approaches in a number of studies. Histological examination has revealed granulomatous inflammation in myotomes and myosepta, frequently containing large vacuoles surrounded by melano-macrophages. T-cells and MHC class II^+^ cells dominate the leukocyte infiltrates in the affected tissues. Nevertheless, myocytes seem to be the primary affected cell type, and therefore the term “chronic polyphasic necrotizing myopathy” has been applied [1,3]. Melano-macrophages are either found in clusters or scattered throughout the changes with melanin produced in situ, making this granulomatous reaction a unique inflammatory response [3]. The characteristic histological appearance indicates that the pigmentation caused by chronic inflammatory response is due to the underlying necrotizing myopathy. The granulomatous inflammation indicates a chronic antigenic stimulation, for instance a foreign-body reaction as suggested before [1] or a chronic infection. However, the aetiology of this myopathy has remained enigmatic. Several viral infections in farmed salmonids are chronic. Both *Piscine orthoreovirus* (PRV) and Salmonid alphavirus (SAV) cause skeletal muscle inflammation in Atlantic salmon [13,14]. PRV-caused changes are most prominent in red muscle, while SAV-caused changes are found in both red and white muscle [13,15]. SAV is widespread in southern parts of the Norwegian Coast and only sporadically found in the northern part. In contrast, melanised focal changes are found in farmed salmon including areas that never have experienced SAV infections.

Heart- and skeletal muscle inflammation (HSMI) is associated with PRV, and is characterized by severe pancarditis, inflammation and necrosis of red myocytes in Atlantic salmon. Pathological changes have also been described in white myocytes [16]. Several PRV-screenings based on reverse transcription qPCR (RT-qPCR) have shown that PRV is almost ubiquitously present in seawater-farmed Atlantic salmon in Norway. PRV has been detected in apparently healthy fish throughout the production cycle [17], and has also been found in wild salmon [18,19]. Erythrocytes have been found to be the central target cells for PRV, implying that the virus can be found in any organ in an infected individual [20]. Infected erythrocytes contain globular inclusions, i.e. viral factories that contain PRV protein and dsRNA [21,22]. The gene expression pattern due to PRV infection in salmon erythrocytes includes large-scale changes of immune regulators [23].

In this study, we examined samples from groups of farmed, wild and in-house Atlantic salmon for the occurrence of red or melanised focal white muscle changes and tested for the presence of different viruses. Here we show large amounts of PRV in such changes.

## Materials and methods

### Groups

The study included eight different fish populations (A-H). Groups A-D were sea-farmed fish collected at different farm sites in west and north on the Norwegian coast. Groups A and B were selected because of high and low prevalence of melanised focal changes, respectively. As red focal changes are much less frequent compared with melanised focal changes, an additional sampling was conducted where only muscle samples were collected on RNA*later* and formalin (Group C). Group D consisted of archived paraffin-embedded material of melanised focal changes sampled in 2008. Groups E-G were fish collected at Institute for Marine Research (Matre Research Station). Group E consisted of vaccinated fish kept in sea cages. Group F was vaccinated fish kept in sea water in-house tanks together with a group of unvaccinated fish (Group G). The sea-water was obtained from 70 meters depth. Group H was wild Atlantic salmon caught with dip-net at Hellefossen in the Drammen River following their return for spawning, approximately 400 km away from the nearest commercial sea-farm. Details for the different groups are presented in Table 1.

**Table 1:** **Group information**

**Group**	**Fish (n)**	**Age (y)**	**Broodstock**	**Water**	**Length (cm)**	**Weight (g)**
A	25	2	Farmed	Sea	n/a	4144
B	35	2	Farmed	Sea	n/a	4056
C	10	2	Farmed	Sea	n/a	4696
D	26	n/a	Farmed	Sea	n/a	n/a
E	20	3	Farmed	Sea	73.5 ± 5.6	4908 ± 1009
F	42	3	Farmed	Sea	60.5 ± 5.5	2763 ± 815
G	42	3	Farmed	Sea	57.4 ± 7.7	2953 ± 747
H	10	n/a	Wild	Fresh*	81 ± 12.5	5020 ± 2210

^*^returning to river.

(n/a - Not available).

Locations: **A** – Northern Coast. **B, C**, **D**, **E, F** and **G** – Western Coast. **H** – Drammen River, South-east.

### Sampling

First, peripheral blood was collected in vacutainer tubes containing heparin. The fish were subsequently bled to death and opened by an abdominal incision. The organs were examined and the fish were filleted and the muscle examined for changes. Fish with and without visible muscle changes were used for the subsequent analysis. Samples from white muscle and spleen were collected on RNA*later* and 10% phosphate-buffered formalin. From individuals where muscle discolouration were detected, either as red or melanised focal changes, samples from affected areas and from macroscopically non-affected muscle were collected as described above. Hearts from all fish in Groups A-B and E-H were collected in formalin. All fish included in this study were anaesthetised prior to sampling, in line with regulations of the Norwegian Directorate of Fisheries.

### Anamnestic information

Groups A-F had been intraperitoneally immunised, while the fish in Group G was left un-vaccinated. No anamnestic information was available for the wild fish (Group H). Groups A-E had been routinely deloused, whilst Groups F-G (kept in in-house tanks) received no such treatments. Group A was diagnosed with severe outbreak of HSMI with 9% mortality 5 months prior to sampling. HSMI had been suspected in Group C, but was not investigated further. Despite being vaccine-naïve, no diseases were recorded in Group G, and no mortality was observed during the observation period.

### Histology and immunohistochemistry

The tissue samples were fixed in formalin for 24–48 h, and next dehydrated and embedded in paraffin according to standard procedures. The slides were cut 2 μm thick and mounted on glass slides (Superfrost®, Mentzel, Braunschweig, Germany), incubated for 24 h at 37 °C, de-waxed in xylene and rehydrated through graded alcohol baths. Sections were stained according to standard procedures with haematoxylin and eosin (HE), with van Gieson’s method for detection of collagen and with Perl’s Berlin blue for detection of ferric iron. The slides were mounted with polyvinyl alcohol media (Ullevål Apotek, Oslo, Norway).

The paraffin-embedded material was also used for immunohistochemical investigations. The different steps were performed at room temperature unless otherwise stated. The sections were cut 4 μm thick and mounted on glass slides (Superfrost©; Mentzel, Braunshweig, Germany), incubated for 24 h at 37 °C and thereafter for 30 min at 58 °C, de-waxed in xylene and rehydrated in graded alcohol baths before transferring to distilled water. Sections were next autoclaved in 0.01 M citrate buffer, pH 6.0 at 120 °C for 10 min to retrieve antigens, followed by treatment with phenylhydrazine (0.05%; Sigma-Aldrich, St. Louis, MO, USA) for 40 min at 37 °C to inhibit endogenous peroxidase. The slides were rinsed three times in phosphate-buffered saline (PBS).

Nonspecific binding was prevented by adding PBS with 2% (v/v) normal goat serum plus 5% (w/v) bovine serum albumin (BSA) or 5% (w/v) skimmed dry milk. The primary antibody –in tris-buffered saline (TBS) with 1% BSA - was added and incubated for 30 min. The sections were then rinsed three times in TBS and, and further incubated with a secondary antibody (EnVision© System kit; Dako, Glosrup, Denmark) for 30 min. The slides were again washed three times in TBS, and the sections were incubated with AEC for 14 min or DAB for 7 min (EnVision© System kit) to evoke, respectively, red or brown colour. Sections were washed with distilled water and counterstained with Mayers hematoxylin for 1 min and mounted with polyvinyl alcohol media pH 8.0. Two different anti-PRV rabbit sera, detecting respectively endosomal membrane penetration protein μ1C and cell attachment protein σ1, were used to identify PRV [14]. Additionally, immunohistochemistry was performed by antibodies against *Infectious pancreatic necrosis virus* (IPNV) [24] and SAV [13]. Pancreas samples from field outbreaks of IPN and pancreas disease (PD) in Atlantic salmon were used as positive controls for the latter two infections (provided by the Norwegian Veterinary Institute, Oslo). Heart samples from Atlantic salmon collected during a challenge study with HSMI were used as positive controls for PRV [20]. Negative controls were performed using 1% BSA instead of the primary antibody and by using rabbit serum collected prior to immunization.

### RNA isolation

Total RNA was isolated from skeletal muscle and spleen samples stored in RNA*later* and from heparinized blood. Briefly, samples from spleen, white muscle (50 mg) and blood (15 μL) were homogenized in QIAzol Lysis Reagent (Qiagen, Hilden, Germany) using 5 mm steel beads and TissueLyser II (Qiagen) for 2 × 5 min at 25 Hz. Chloroform was added, samples were centrifuged and the aqueous phase collected. RNA was isolated using RNeasy Mini QIAcube Kit (Qiagen) according to manufacturer’s instructions. RNA was quantified using NanoDrop ND-1000 spectrophotometer (Thermo Fisher Scientific, Wilmington, DE, USA).

### RT-qPCR for viral RNA

Blood and skeletal muscle samples were screened for PRV by RT-qPCR using Qiagen OneStep kit (Qiagen) targeting PRV segment S1 as previously described [20]. A standard input of 100 ng (5 μL of 20 ng/μL) from the isolated total RNA was used in each reaction. The numbers of blood samples tested from each group were: A (*n* = 25), B (*n* = 35), E (*n* = 20), F (*n* = 42), G (*n* = 42) and H (*n* = 10), and for sample pairs of white skeletal muscle from changed and non-affected areas: Group A (*n* = 9), C (*n* = 6), E (*n* = 14), and non-affected white skeletal muscle from groups without changes; i.e. Group F (*n* = 6) and G (*n* = 6).

Six spleen samples from each group, apart from Groups C (only muscle sampled) and D (only paraffin-embedded material), were analysed for other viruses and screened by RT-qPCR for IPNV, infectious salmon anemia virus (ISAV), piscine myocarditis virus (PMCV), SAV and viral hemorrhagic septicemia virus (VHSV). Reverse transcription was performed using Superscript III Reverse Transcriptase (Invitrogen, Carlsbad, CA, USA) according to the manufacturer’s instructions with 1000 ng RNA input per sample. All qPCR analysis were run in duplicate in Mx3005P (Stratagene, La Jolla, CA, USA) using cDNA corresponding to 15 ng RNA input. The qPCR for detection of IPNV, ISAV, SAV and VSHV was performed with Taqman Universal PCR master mix (Applied Biosystems) including 300 nM primer and 200 nM probe in a 13 μL reaction volume. The following cycling conditions was used: 50 °C/2 min, 95 °C/10 min, 40 cycles of 95 °C/15 s, 58 °C/15 s and 60 °C/60 s. Elongation factor 1αβ (EF1αβ) was used as reference gene. For detection of PMCV, MESA Blue qPCR Mastermix Plus for SYBR assay (Eurogentec, Liège, Belgium) was used with primer concentration of 400 nM in a 15 μL reaction volume. The following cycling conditions was applied; 95 °C/5 min, 40 cycles of 95 °C/15 s and 54 °C/60 s. Primers and probes used in this study are listed in Table 2 [20,25-30]. The primers and probe used for SAV pick up all subtypes of SAV, including the subtypes currently found in Norway, i.e. SAV2 and SAV3.

**Table 2:** **Nucleotide sequences of primers and probes used in this study**

**Target**	**Primer/probe**	**Sequence (5’ → 3’)**	**Reference**
PRV	S1 Fwd	TGCGTCCTGCGTATGGCACC	[20]
	S1 Rev	GGCTGGCATGCCCGAATAGCA	
	S1 Probe	ATCACAACGCCTACCT	
SAV	nsP1-Fwf	CCGGCCCTGAACCAGTT	[27]
	nsP1-Rev	GTAGCCAAGTGGGAGAAAGCT	
	nsP1-Probe	CTGGCCACCACTTCGA	
ILAV	S7 Fwd	TGGGATCATGTGTTTCCTGCTA	[30]
	S7 Rev	GAAAATCCATGTTCTCAGATGCAA	
	S7 Probe	CACATGACCCCTCGTC	
IPNV	VP3 Fwd	CGACCGACATGAACAAAATCA	[28]
	VP3 Rev	AGTTGCAGCTGTATTCGCACA	
	VP3 Probe	TCTAGCCAACAGTGTGTACGGCCTCCC	
VHSV	N Fwd	GACTCAACGGGACAGGAATGA	[29]
	N Rev	GGGCAATGCCCAAGTTGTT	
	N Probe	TGGGTTGTTCACCCAGGCCGC	
PMCV	Fwd	TTCCAAACAATTCGAGAAGCG	[25]
	Rev	ACCTGCCATTTTCCCCTCTT	
EF 1αβ	Fwd	TGCCCCTCCAGGATGTCTAC	[25]
	Rev	CACGGCCCACAGGTACTG	
	Probe	AAATCGGCGGTATTGG	

The data was analysed by MxPro v4.10 (Stratagene). A sample was defined as positive if both parallel samples had a Ct < 35. The PRV RT-qPCR results from blood and skeletal muscle samples were used to calculate the mean Ct value and SD for each group. Ct value for samples were no Ct value was found was set to 35. Statistical analyses of differences were done by Wilcoxon matched pairs signed rank test using GraphPad Prism (GraphPad Software inc., USA), and p-values of *p* ≤ 0.05 were considered as significant.

## Results

### Macroscopic examination

**Figure 1:**
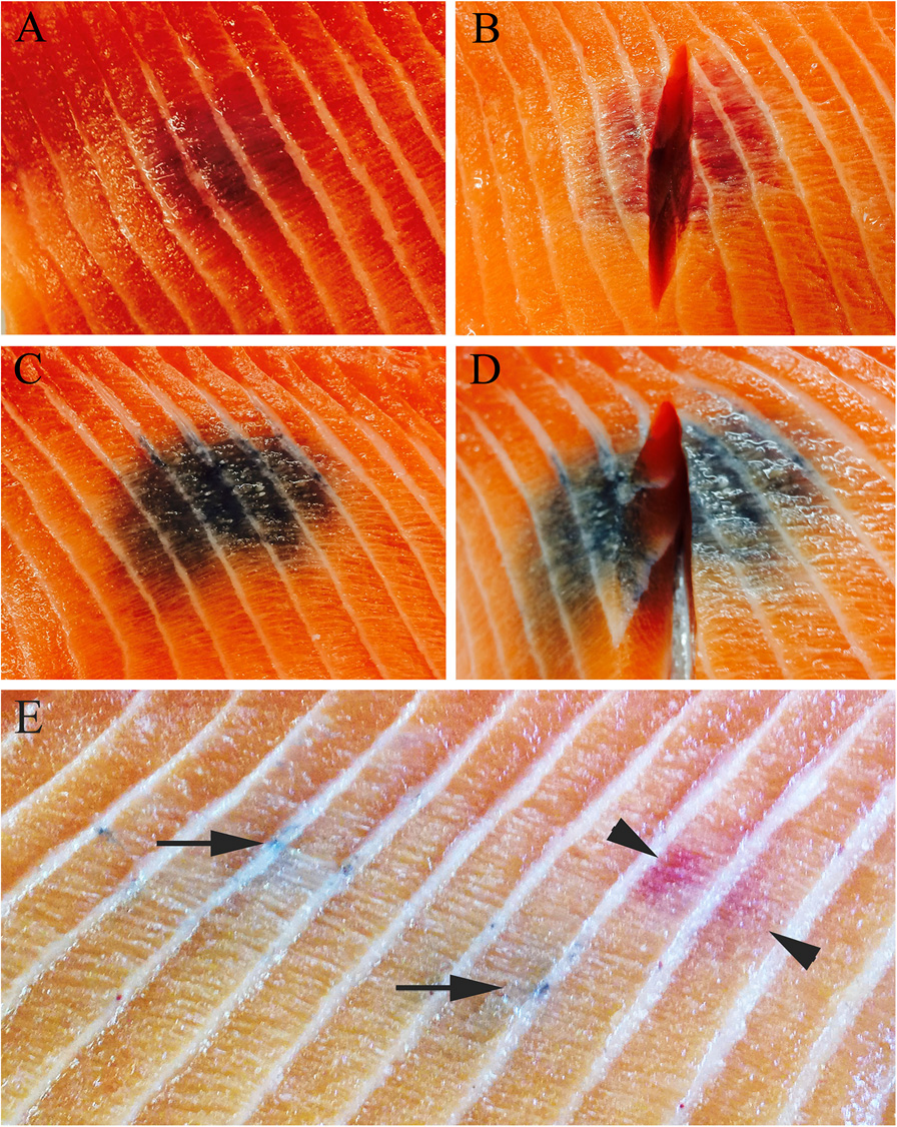
**Gross pathological changes of white muscle. (A)** A red focal change in the muscle of the craniodorsal region of the abdominal wall. **(B)** An incision through a red focal change showing discolouration extending deep into the fillet. **(C)** A melanised focal change detected in the same anatomical location in a different fish. **(D)** A melanised focal change with extensive amounts of a whitish tissue extending into the depth of the fillet. **(E)** A muscle fillet with two faint melanised lesions (arrows) and one red focal change (arrowheads).

All fish of all groups were in normal condition and with no apparent external pathological changes. Following autopsy, large discoloured focal changes in the white muscle, mainly in the cranio-dorsal and abdominal regions, were found in Groups A, B, C, D and E, but not in Groups F, G and H, including fish kept in in-house tanks and in wild-caught individuals (Figure 1 and Table 3). One faintly pigmented focus, approximately 2 mm in diameter, was detected in one individual in Group F. This change would have gone unnoticed under normal abattoir conditions.

**Table 3:** **Macroscopic distribution of red and melanised focal changes**

**Group**	**Fish (n)**	**Melanised changes (n)**	**Red changes (n)**	**No changes (n)**	Heart changes* (n)
A	25	9	1	15	20
B	35	1	0	34	5
C	10	3	7	0	n/a
D	26	26	0	0	n/a
E	20	17	1	2	15
F	42	1	0	41	0
G	42	0	0	42	0
H	10	0	0	10	0

^*^Detection of focal or multi-focal endocarditis, myocarditis or epicarditis.

(n/a - Not available).

The lesions in the white muscle were macroscopically classified as either red or melanised focal changes.

**Figure 2:**
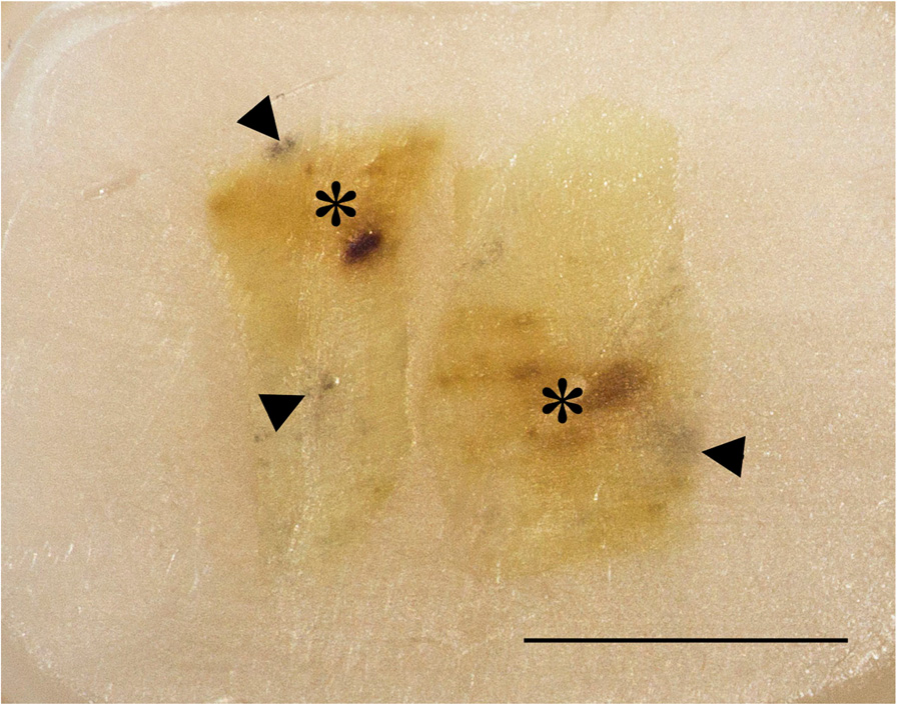
**Gross image of paraffin-embedded red focal change.** Transient form with both red (asterisk) and melanised (arrowheads) changes. Scale bar = 1 cm.

In general, each affected fillet had only one focal change, but occasionally there were multiple foci with varying degrees of discolouration within a single focal change. Foci extended from 1–3 cm and could involve 2–6 myotomes. Incisions through foci revealed extension of the discolouration deep into the muscle (Figures 1B and D). The changes generally appeared as either red or melanised (Figure 1 and Table 3). Two changes classified as red contained both red and melanised discolouration (Figure 2).

### Histological examination

#### Red focal changes

**Figure 3:**
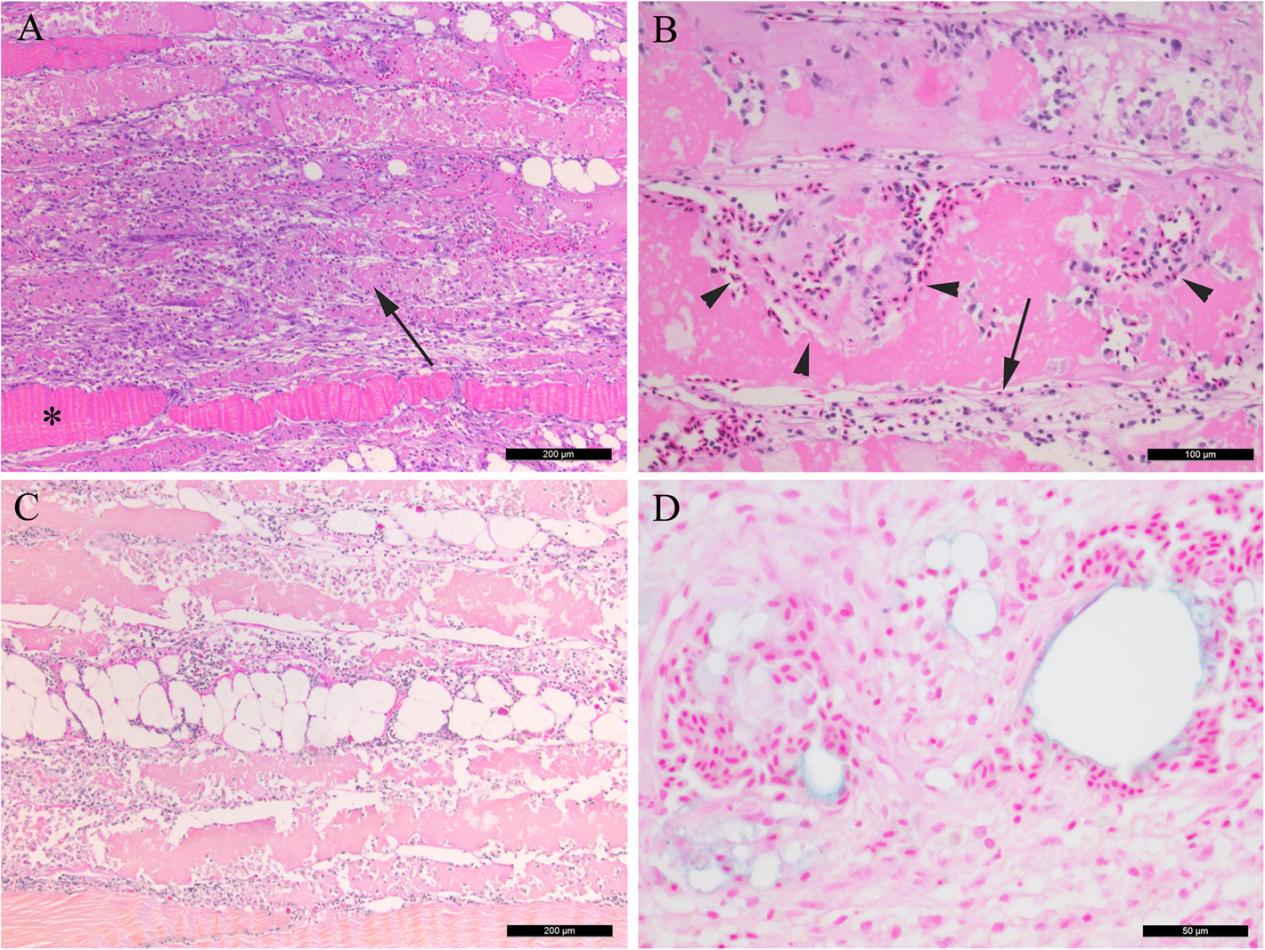
**Histological investigations of red focal changes. (A)** Necrotic myocytes display pale cytoplasm and appear homogenous without striations (arrow). Myocyte with striations (asterisk) (HE staining). **(B)** Necrotic myocytes with erythrocytes apparently located within the myocytes (arrowheads) and some infiltrations of macrophage-like cells (arrow) (HE staining). **(C)** Centrally located vacuoles of varying size, surrounded by moderate levels of collagen (intense red) and modest numbers of leukocytes (van Gieson staining). **(D)** Focal haemorrhage surrounding vacuoles. The edges of the vacuoles are positive for ferric iron (blue colour) (Perls’ Berlin blue staining). **(A, C)** scale bar = 200 μm, **(B)** scale bar = 100 μm, **(D)** scale bar = 50 μm.

Red focal changes in individuals of Groups A, C and E were all characterised by an acute haemorrhagic necrotic myositis. In more detail, we observed severely disorganised architecture of tissues including several myocytes with a complete loss of striation conformal with coagulation necrosis (Figure 3A). Organisation into granulomas was not observed. Poikilocytosis was highly prevalent, as both elongated and rounded erythrocytes were present. Focal areas of haemorrhage and accumulation of extravasal erythrocytes were present in all red changes accompanied by coagulation necrosis of myocytes. Erythrocytes and macrophage-like cells were observed both between and within necrotic myocytes (Figure 3B). In some changes, myocytes adjacent to the affected site showed signs of regeneration displaying a basophilic appearance and a central rowing of nuclei. Occasional melano-macrophages were detected. Fibrosis was present to some degree, mainly associated with vacuoles that varied in size and shape (Figure 3C). Staining with Perls’ Berlin blue technique confirmed the presence of haemosiderin in the periphery of the vacuoles, especially in association with large infiltrates of erythrocytes (Figure 3D).

In the two changes characterised as red with some visible melanisation (Figure 2), the histological picture concurred with the description above, whereas the findings in the melanised portion was consistent with the description of the melanised focal changes (below).

#### Melanised focal changes

**Figure 4:**
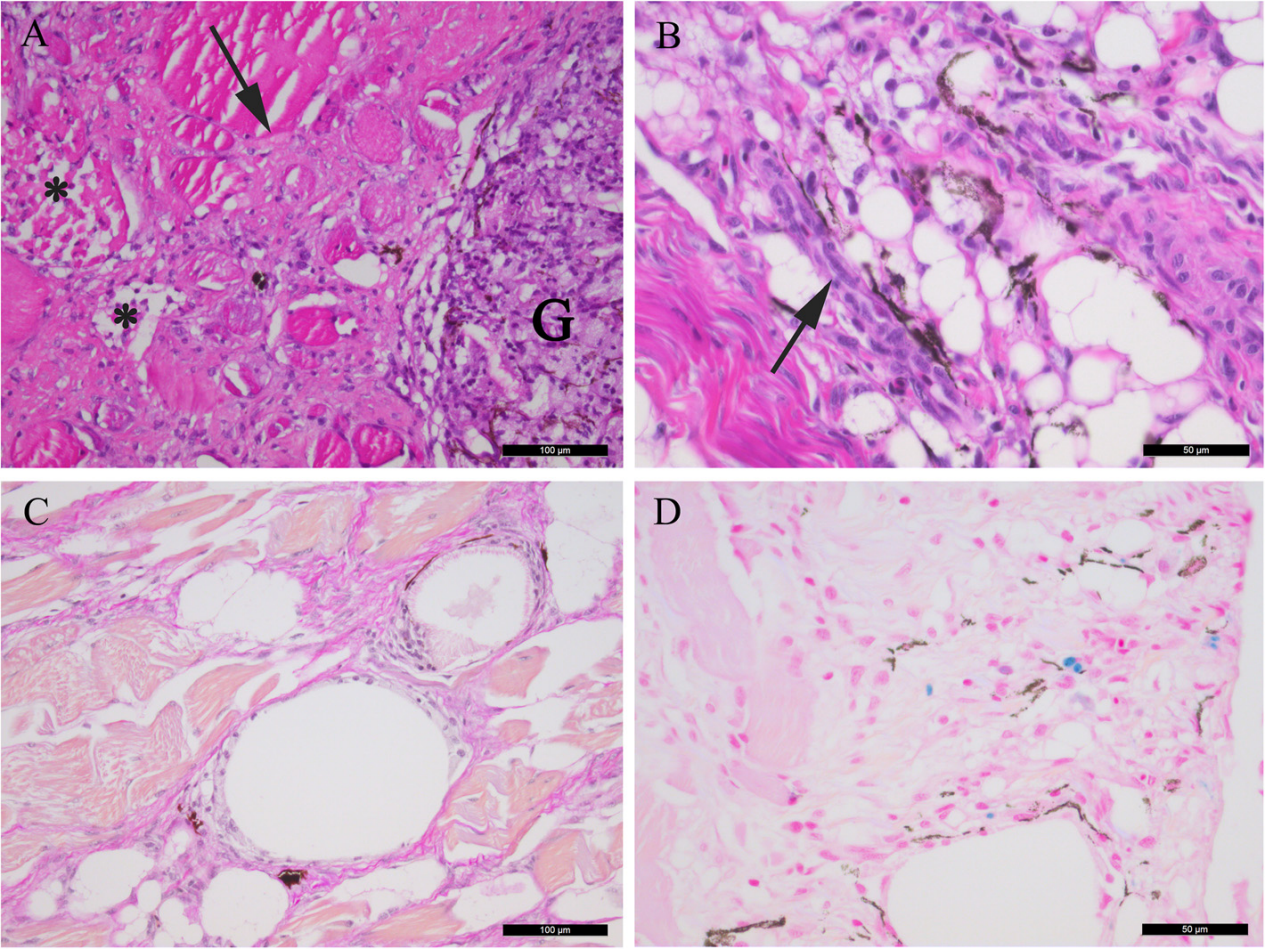
**Histological investigations of melanised focal changes. (A)** Transverse section of necrotic muscle cells (asterisk) and severe fibrosis (arrow) with infiltrates of leukocytes. A cell-rich granuloma with melanin-containing cells is present in the right part of the picture (G), displaying a heterogeneous morphology (HE staining). **(B)** Severe vacuolisation and adipocytes with surrounding melano-macrophages (black) and clusters of regenerating myocytes with a basophilc cytoplasm (arrow) (HE staining). **(C)** Multiple vacuoles in an area with severe fibrosis (intense red staining) (van Gieson staining). **(D)** Several iron-containing macrophage-like cells (blue staining) in association with melano-macrophages (black) (Pearls’ Berlin blue staining). (A, C) scale bar = 100 μm, (B, D) scale bar = 50 μm.

Melanised focal changes in individuals of Groups A-F were characterised by chronic degenerative inflammation with well-organised granulomas. This included central severe fibrosis, but also peripheral acute extensive tissue destruction and necrosis. Melano-macrophages were abundant throughout the granulomatous tissue and within well-organised granulomas (Figure 4A). Vacuoles were observed throughout the affected areas (Figure 4B). Signs of repair with extensive fibrosis and regeneration were detected in the chronically inflamed lesions (Figure 4C), confirming previous tissue destruction and subsequent repair. Haemorrhages were not observed. Macrophages with hemosiderin were detected in association with melano-macrophages, though only sparse amounts of positive cells were observed (Figure 4D). Group F contained one individual with a very faint pigmented spot, and examination of this sample showed muscle necrosis and some inflammatory infiltrates containing melano-macrophages.

#### Hearts

**Figure 5:**
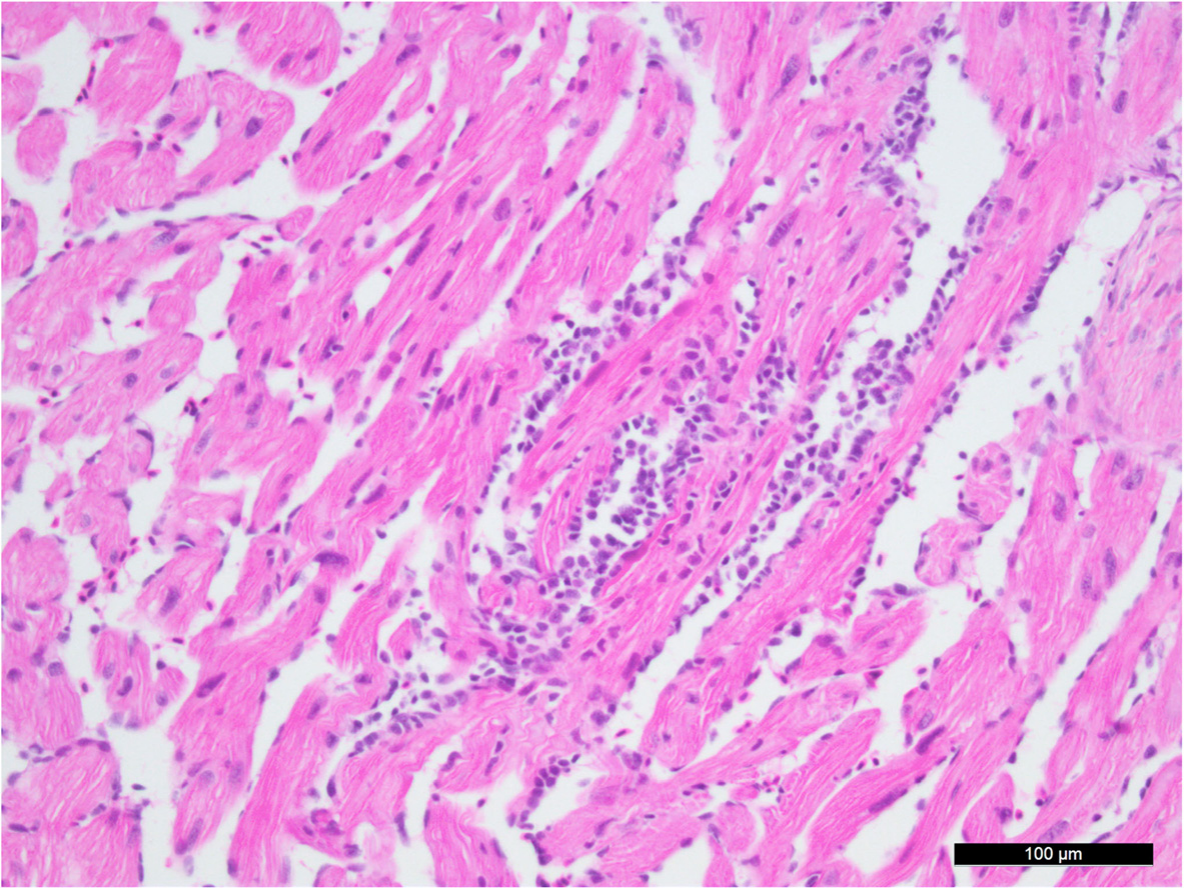
**Histological image of cardiac spongious layer.** Infiltrates of leukocytes are visible in endocard and myocard. Scale bar = 100 μm.

In Groups A, B and E, moderate endocarditis, myocarditis (Figure 5) and epicarditis conformal with HSMI-related changes were detected (Table 3). Focal infiltrates of leukocytes were observed in either the compact or the spongious layers or both, and sometimes inflammatory foci were seen in the atrium. Degeneration of muscle fibres was not detected. No changes were observed in the fish kept in in-house tanks or in the wild fish (Groups F-H).

### Immunohistochemistry for PRV

All focal white muscle changes in Group A-F were investigated and found positive for PRV-antigen. In general, the staining intensity was most prominent in acute compared to chronic stages, as assessed by the degree of organisation and content of connective tissue.

#### Red focal changes

**Figure 6:**
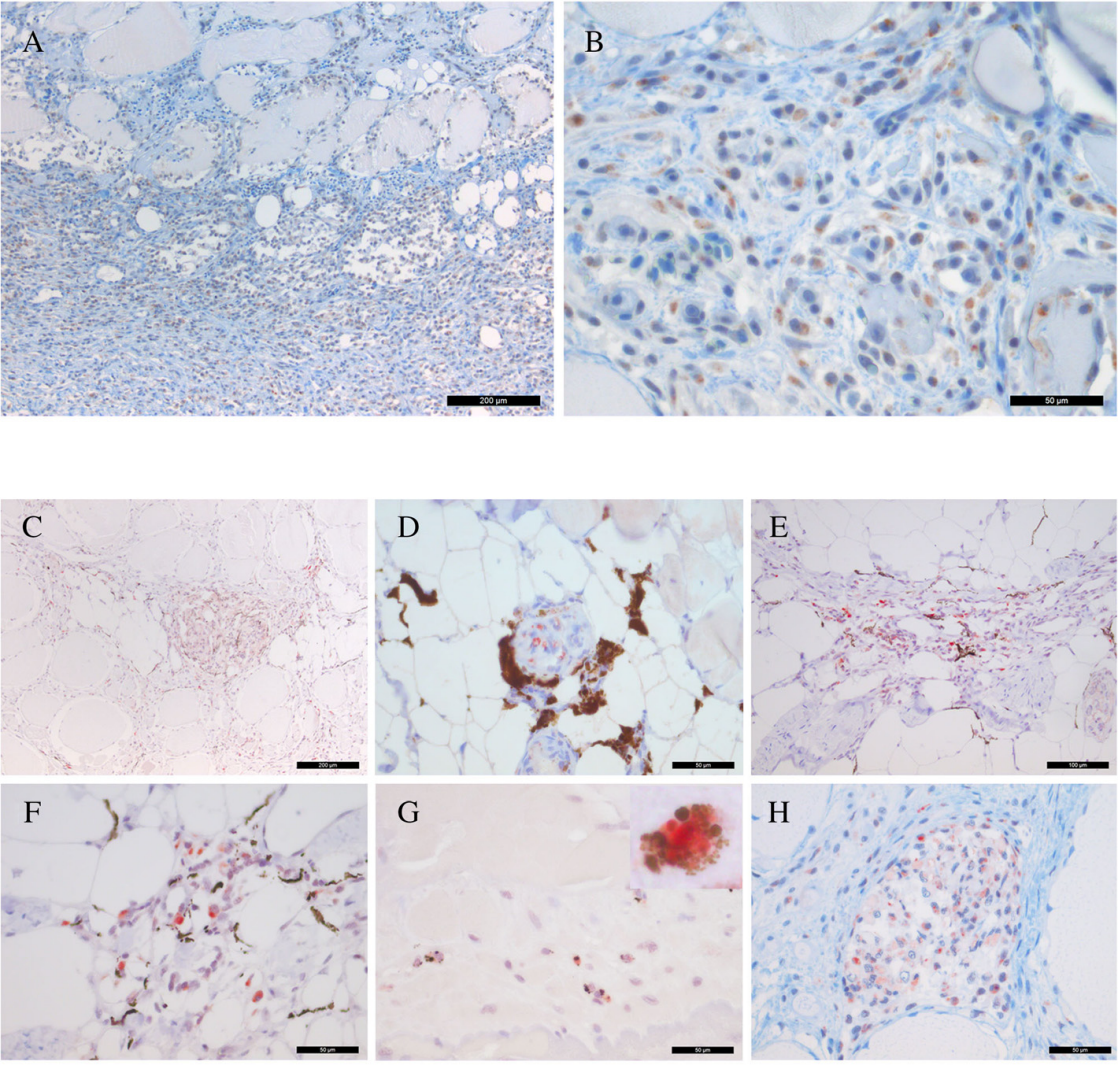
**Immunohistochemical staining for PRV antigens of red (A-B) and melanised (C-H) focal changes. (A)** Abundant amounts of PRV-positive cells (brown) and erythrocytes in the necrotic muscle tissue. **(B)** Transverse section of necrotic myocytes with intracellular PRV-positive macrophage-like cells and erythrocytes (brown). Distinct granular staining is present in the cytoplasm of the macrophage-like cells. **(C)** A well-organised granuloma is present in the center of the image, heavily positive for PRV (red), and with vast amounts of elongated melano-macrophages (black). **(D)** A single granuloma with PRV-positive cells (red) surrounded by heavily pigmented melano-macrophages. **(E)** A focus with necrotic tissue and infiltrates of melano-macrophages (black) and macrophage-like cells positive for PRV (red). **(F)** A close-up of E where the distinct reaction in the cytoplasm is evident (red). **(G)** PRV antigen (red) in melano-macrophages (black) in a necrotic myocyte. Higher resolution image in the upper right corner (100 x). **(H)** A single necrotic myocyte undergoing phagocytosis and containing abundant PRV-positive macrophage-like cells (red). (A, C) scale bar = 200 μm, (E) scale bar = 100 μm, (B, D, F, G, H) scale bar = 50 μm

Large amounts of PRV antigens were found in macrophage-like cells and in erythrocytes in the haemorrhagic necrotic tissue (Figure 6A). PRV-antigen positive cells were observed both outside and within necrotic myocytes. Myophagocytosis was highly prevalent, with substantial number of PRV-antigen positive macrophage-like cells within myocytes. Staining was cytoplasmic even in most PRV-antigen positive cells, though cells also displayed polar granular staining (Figure 6B). Such cytoplasmic PRV inclusions have previously been identified with confocal microscopy and immunofluorescent staining as viral factories [20].

#### Melanised focal changes

Immunohistochemistry from all affected groups, including the single individual in Group F, revealed intense reactivity against PRV in granulomas and in mononuclear cells (Figures 6C and D). Positive macrophage-like cells were scattered throughout the chronically inflamed areas. In PRV-antigen positive cells staining was observed predominantly as cytoplasmic inclusions (Figures 6E and F). Melano-macrophages positive for PRV were occasionally detected in necrotic myocytes (Figure 6G), but in general they were infiltrated with PRV-positive macrophage-like cells (Figure 6H). A macroscopically faint discolouration found in one individual revealed scattered necrotic myocytes containing PRV-antigen positive macrophage-like cells and occasional melano-macrophages. The two different anti-PRV sera, detecting respectively capsid protein μ1C and σ1, both had similar staining patterns.

#### Controls

In the PRV- RT-qPCR negative control group (Group H), no changes or PRV staining were detected. Control white-muscle samples from fish with red or melanised focal changes were all PRV-antigen negative unless necrosis was present. When necrosis was present, i.e. without macroscopically red or melanised changes, the myocytes contained PRV-antigen-positive macrophage-like cells. A similar occurrence of positive cells in degenerated muscle cells was also seen in occasional fish without focal white muscle changes. No staining was detected when the primary antiserum was omitted or replaced with the pre-immune serum. Six fish from each group were further investigated for the presence of IPNV [24] and SAV [13] with immunohistochemistry. All fish were negative.

### PRV detection by RT-qPCR

#### PRV load in blood

**Figure 7:**
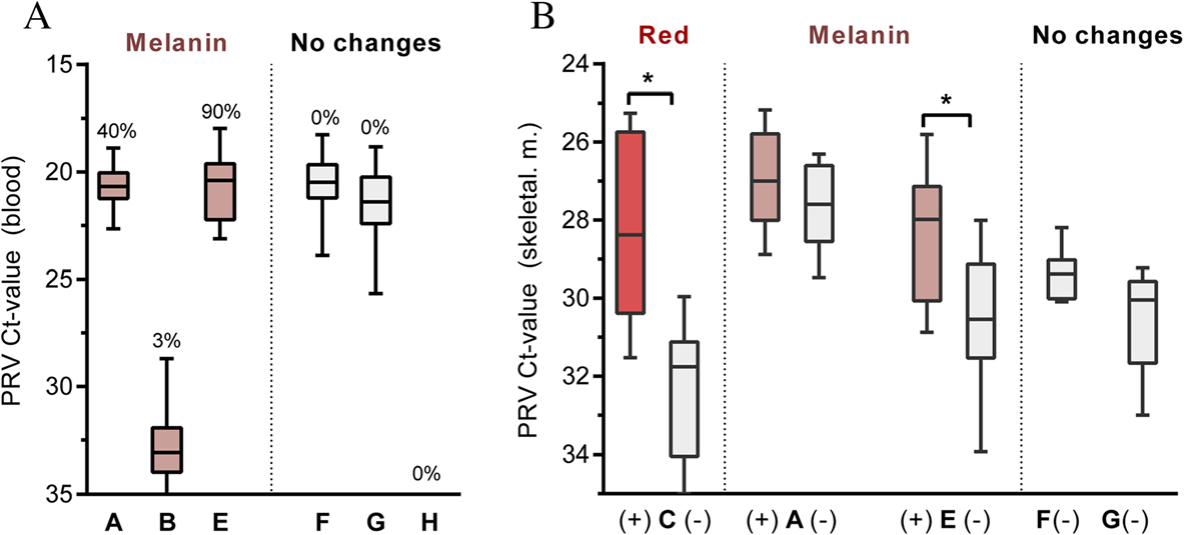
**RT-qPCR analysis for PRV in blood (A) and white skeletal muscle (B).** Fish groups with melanised, red or no changes are coloured brown, red, and grey, respectively. (A) Detection in blood presented by box plot with whiskers indicating max/min of the Ct-values from Group A (*n* = 25), B (*n* = 35), E (*n* = 20), F (*n* = 42), G (*n* = 43) and H (*n* = 10). The percent of fish in each group with melanised focal changes are displayed. (B) Samples from red or melanised focal changes (+) and non-affected areas (−) from the same individual. Box plot and whiskers showing max/min of the Ct-values from Group C with red focal changes (*n* = 6), and from Groups A (*n* = 9) and E (*n* = 14) with melanised focal changes. Only non-affected tissue (−) could be tested from groups without changes; i.e. Groups F (*n* = 6) and G (*n* = 6). Analyses were performed using Wilcoxon matched pairs signed rank test. *p < 0.05.

The PRV loads in blood samples of Groups A, B, E-H were assessed by RT-qPCR (Figure 7A). All groups in which melanised changes were found (Groups A, B, E) contained RT-qPCR PRV positive fish. High PRV loads were detected in Group A (Ct 20.7 ± 0.8) and Group E (Ct 20.7 ± 1.4), in which prevalences of melanised changes were 40% and 90%, respectively. The viral load was much lower in Group B (Ct 32.8 ± 1.7) where melanised changes were much less prevalent (3%). High levels of PRV were also detected in Group F (Ct 20.6 ± 1.2) and Group G (Ct 21.4 ± 1.4) where melanised focal changes were absent. The wild fish of Group H were the only group negative for both PRV and melanised changes. Overall, the load of PRV in blood was not correlated with the presence of melanised changes.

#### PRV load in red and melanised changes

The PRV loads in samples from red and melanised changes were assessed by RT-qPCR and compared to samples collected from non-affected white muscle tissue of the same individual fish (Figure 7B). The red changes, observed in Group C, contained a significantly higher viral load compared to control tissue with Ct values 28.2 ± 2.4 and 32.3 ± 1.8, respectively (*p* < 0.05). The viral loads in melanised changes from Group E were also significantly higher than that of the control (Ct 28.3 ± 1.5 and Ct 30.4 ± 1.6) (*p* < 0.05), while no significant difference was found from group A. White muscle tissues from fish with no spots in Groups F and G had a Ct value of 29.4 ± 0.7 and 30.5 ± 1.4, respectively. The viral loads observed in the white muscle tissues (Figure 7B) were significantly lower than that observed in blood (Figure 7A).

#### Controls

The six spleen samples from each of Group A, B, E-H were all negative for IPNV, ISAV, PMCV, SAV and VHSV by RT-qPCR.

## Discussion

In this study, we investigated red and melanised focal changes as well as transient, partly red and melanised changes in white muscle of Atlantic salmon. Histological investigations revealed that red focal changes were dominated by haemorrhages and myocyte necrosis, consistant with acute manifestations of muscle damage. The focal melanised changes were dominated by granulomatous tissue rich in collagen, indicating chronic inflammatory response. In these changes, melano-macrophages were abundant, and iron-containing macrophage-like cells were observed, indicating previous haemorrhage. The transient forms displayed both red (acute) and melanised (chronic) changes as described above. Macroscopic observations showed that all forms may occur in the same individual simultaneously. Together, these results indicate that the melanised focal changes arise as a consequence of chronic inflammation.

The common denominator to all red and melanised changes was the presence of large amounts of PRV antigens detected by immunohistochemistry. In red focal changes, i.e. acute manifestations, PRV antigen was found in erythrocytes and macrophage-like cells in necrotic myocytes. In addition, the viral loads as measured by RT-qPCR, were significantly higher in red focal changes than in surrounding non-affected muscle. In melanised changes, i.e. chronic changes, PRV-antigen was detected in macrophage-like cells and melano-macrophages within granulomas and in less organised granulomatous tissue. Our findings indicate that a focal PRV infection is a premise for the transition from red to melanised focal changes, and the lack of capability to eliminate PRV or PRV-antigen is the driving power behind this process.

The results of immunohistochemistry clearly linked PRV to the focal lesions. Several approaches unanimously demonstrated the specificity of the PRV immunostaining. Two different PRV rabbit antisera both displayed cytoplasmic staining of macrophage-like cells within the white muscle changes. Samples from areas without red and melanised focal changes from affected or non-affected fish were in most cases negative, but macrophage-like cells in solitary degenerated myocytes were positive. No other viruses were detected in any of the groups. One group of wild salmon from a river located far away from commercial farming was included in the study (Group H). The fish were found to be PRV negative in blood by RT-qPCR, negative for melanised focal changes by visual examination, and PRV-antigen negative by immunohistochemistry. As both PRV and melanised focal changes are prevalent in farmed Atlantic salmon, it was imperative for this study to include such negative control material in the investigations.

Erythrocytes are important target cells for PRV [19,21]. In our study positive staining for PRV was also found in macrophage-like cells and melano-macrophages. We do not know if PRV replicates in all these different cell types. Replication of PRV takes place in cytoplasmic structures called viral factories, which appear as dense inclusions [20]. Intracellular structures with dense staining that may resemble viral factories were prevalent findings in the red and melanised focal changes. This is in line with an on-site propagation of PRV. The amount of PRV RNA, as assessed by Ct values from red focal changes was significantly higher than from corresponding non-affected muscle tissues from the identical individual. This correlation was not fully consistent for melanised changes. This indicates that replication of PRV occurs at the sites with red changes, while PRV-antigen persists into the formation of granuloma. Although macrophages engulf viruses to inactivate them, many viruses are able to replicate in macrophages [31-33]. This may provide a long-term shielded environment for viral replication, and the process will attract more macrophages and culminate in a chronic inflammatory response. Comparatively, avian orthoreoviruses (ARV) viral antigen and RNA have been documented in macrophages [34]. ARV are ubiquitous in avian farming and belong to the same genus as PRV, and virulent ARV strains have shown enhanced ability to replicate in macrophages [35].

PRV replicates in erythrocytes and therefore virus loads were assessed in blood samples. All groups with focal muscle changes were positive for PRV by RT-qPCR in blood. However, similar viral loads were found in groups without changes. The general PRV load in a fish, as mirrored by the amount in blood, is thus not predicative for the presence of focal muscle changes. This indicates that PRV infection by itself is not sufficient to induce melanised focal changes in the white muscle and that environmental and management factors may be of importance. We cannot exclude that the condition may be initiated by for example trauma. However, melanisation is an insignificant problem in rainbow trout farmed under similar conditions with similar handling and risk of trauma. It could be mentioned that also HSMI, which is prevalent in farmed Atlantic salmon, is negligible in farmed rainbow trout.

High loads of PRV in blood were detected by RT-qPCR in land-based Groups F and G where the fish were almost devoid of macroscopically observable muscular changes. The average weight of the fish in these groups was approximately 2.8 kg while that of the other groups of farmed fish were 4.4 kg. It should not be ruled out that development of the changes is more frequent late in the production cycle. Another possibility is that the PRV strain in Groups F and G was less virulent or the strain of fish could be less susceptible, but this is not supported by the fact that the fish was kept and raised at the same farm as Group E. The almost complete lack of melanisation in Groups F and G indicates that the difference in occurrence of melanisation was related to factors in the management of the populations. In a previous study, genetic constitution, vaccination status and smolification regime were addressed with respect to the frequency of melainised focal changes [10]. Higher frequency was detected in triploid versus diploid fish and in fish smoltified at elevated temperatures after vaccination. The lack of focal muscle changes in the land-based Groups F and G, vaccinated or non-vaccinated, supports previous findings suggesting that vaccination is not a causative factor in the pathogenesis [10-12].

In the present study, the red and melanised focal changes were observed in white muscle. Classically, the muscle lesions related to HSMI are detected in the red muscle and myocardium. However, degeneration and inflammation in white muscle have also been reported in fish with HSMI [16]. HSMI is mainly found during the first months after sea transfer. In contrast, melanised focal changes are predominantly observed at slaughter, indicating a long-term sub-clinical inflammation. The knowledge on long-term effects of a PRV-infection on white muscle is limited. HSMI and focal melanised changes should be considered as two separate conditions; both associated with PRV, but do not necessarily appear simultaneously.

Large amounts of PRV antigens were found in all red and melanised focal changes in salmon groups widely distributed both in time and in geographical locations. The samples from aquaculture used in this study originated from different farm sites west and north on the Norwegian coast, including areas in which SAV infection or the associated Pancreas disease have not been reported. This suits well with the involvement of a ubiquitous aetiological agent such as PRV. Furthermore, the problem with melanised focal changes containing PRV has existed for many years, as demonstrated by the positive results in the archived material (Group D).

Melaninsation contributes to encapsulation and prevents dissemination of intruding pathogens, this being a prominent immune response in arthropods. Such responses therefore have functions similar to those of granulomas in vertebrates. Melanisation possibly also aids the healing process in arthropods. Reactive oxygen species generated during melanisation is thought to be toxic to pathogens, but also to the arthropod host, causing a strict localisation of melanin to the site of inflammation [36]. Our understanding of the function of melanin in the piscine immune responses is in its infancy. Chronic inflammation in fish may appear with abundant pigmentation, as has been found in the peritoneal cavity after deposition of oil-adjuvanted vaccines [1,8]. In our study virus antigen was found at sites with granulomatous inflammation, sometimes encapsulated in well-advanced granulomas.

Our findings indicate that PRV is a premise for the progression of red to the chronic melanised focal changes; however, it does not establish that PRV initiates the process leading to the formation of red focal changes. When a focal PRV infection is established, either as an infection of the myocytes or through infiltrating erythrocytes or macrophage-like cells, the immune response is not able to eliminate the infection to stop the formation of granuloma and melanisation. Myocytes may have been virus-infected prior to necrosis; however, we have no indication for this assumption. Haemorrhage may have developed as a consequence of myocytic necrosis. PRV antigen was found within encapsulated granuloma, the latter being the hallmark of an immune response where the host immobilises and walls off a persistent intruder. Melano-macrophages are known for their participation in such reactions in fish and their presence accounts for the discolouration found in the melanised focal changes. As no melanised focal changes were identified that were devoid of PRV antigen, we believe this presence to be the premise for the prolonged and non-dissolving granulomatous reaction seen in melanised focal changes.

In conclusion, the transition from acute red to chronic melanised changes was justified. Both red and melanised focal changes contain large amounts of PRV antigens, however, environmental and management factors may be pivotal for the initial development of these changes. We suggest PRV to be associated with red and melanised focal white-muscle changes in Atlantic salmon.

## References

[1] Koppang EO, Haugarvoll E, Hordvik I, Aune L, Poppe TT (2005) Vaccine-associated granulomatous inflammation and melanin accumulation in Atlantic salmon, *Salmo salar L.* white muscle. J Fish Dis 28:13–22 10.1111/j.1365-2761.2004.00583.x 15660789

[2] Mørkøre T, Heia K (2012) Black spots in salmon fillet - extent and methods of measurement. Norsk fiskeoppdrett 3:50–53

[3] Larsen HA, Austbø L, Mørkøre T, Thorsen J, Hordvik I, Fischer U, Jirillo E, Rimstad E, Koppang EO (2012). Pigment-producing granulomatous myopathy in Atlantic salmon: a novel inflammatory response. Fish Shellfish Immunol.

[4] Roberts RJ, McQueen A, Shearer WM, Young H (1973). The histopathology of salmon tagging. J Fish Biol.

[5] Haugarvoll E, Thorsen J, Laane M, Huang Q, Koppang EO (2006). Melanogenesis and evidence for melanosome transport to the plasma membrane in a CD83 teleost leukocyte cell line. Pigment Cell Res.

[6] Thorsen J, Høyheim B, Koppang EO (2006). Isolation of the Atlantic salmon tyrosinase gene family reveals heterogenous transcripts in a leukocyte cell line. Pigment Cell Res.

[7] Agius C, Roberts RJ (2003). Melano-macrophage centres and their role in fish pathology. J Fish Dis.

[8] Poppe TT, Breck O (1997). Pathology of Atlantic salmon *Salmo salar* intraperitoneally immunized with oil-adjuvanted vaccine. A case report. Dis Aquat Organ.

[9] Sichel G, Scalia M, Mondio F, Corsaro C (1997). The amphibian Kupffer cells build and demolish melanosomes: an ultrastructural point of view. Pigment Cell Res.

[10] Larsen HA, Austbø L, Nødtvedt A, Fraser TW, Rimstad E, Fjelldal PG, Hansen T, Koppang EO (2014). The effect of vaccination, ploidy and smolt production regime on pathological melanin depositions in muscle tissue of Atlantic salmon, *Salmo salar L*. J Fish Dis.

[11] Berg A, Yurtseva A, Hansen T, Lajus D, Fjelldal PG (2012). Vaccinated farmed Atlantic salmon are susceptible to spinal and skull deformities. J Appl Ichtyol.

[12] Berg A, Bergh Ø, Fjelldal PG, Hansen T, Juell JE, Nerland A (2006) Dyrevelferdsmessige konsekvenser av vaksinasjon av fisk - effekter og bivirkninger (Animal welfare consequences of vaccination of fish - effects and side effects). Institute of Marine Research 1–45

[13] Moriette C, LeBerre M, Boscher SK, Castric J, Bremont M (2005). Characterization and mapping of monoclonal antibodies against the Sleeping disease virus, an aquatic alphavirus. J Gen Virol.

[14] Finstad ØW, Falk K, Løvoll M, Evensen Ø, Rimstad E (2012). Immunohistochemical detection of piscine reovirus (PRV) in hearts of Atlantic salmon coincide with the course of heart and skeletal muscle inflammation (HSMI). Vet Res.

[15] McLoughlin MF, Graham DA (2007). Alphavirus infections in salmonids--a review. J Fish Dis.

[16] Kongtorp RT, Taksdal T, Lyngøy A (2004). Pathology of heart and skeletal muscle inflammation (HSMI) in farmed Atlantic salmon *Salmo salar*. Dis Aquat Organ.

[17] Løvoll M, Alarcon M, Bang Jensen B, Taksdal T, Kristoffersen AB, Tengs T (2012). Quantification of piscine reovirus (PRV) at different stages of Atlantic salmon *Salmo salar* production. Dis Aquat Organ.

[18] Garseth AH, Fritsvold C, Opheim M, Skjerve E, Biering E (2013) Piscine reovirus (PRV) in wild Atlantic salmon, Salmo salar L., and sea-trout, *Salmo trutta L.* in Norway. J Fish Dis 36:483–493 10.1111/j.1365-2761.2012.01450.x 23167652

[19] Garseth AH, Madhun AS, Biering E, Isachsen CH, Fiksdal I, Einen ACB, Barlaup B, Karlsbakk E (2015). Annual report on health monitoring of wild anadromous salmonids in Norway, Norwegian Veterinary Institute and The Institute of Marine Research.

[20] Finstad ØW, Dahle MK, Lindholm TH, Nyman IB, Løvoll M, Wallace C, Olsen CM, Storset AK, Rimstad E (2014). Piscine orthoreovirus (PRV) infects Atlantic salmon erythrocytes. Vet Res.

[21] Wessel Ø, Olsen CM, Rimstad E, Dahle MK (2015). Piscine orthoreovirus (PRV) replicates in Atlantic salmon (*Salmo salar L.*) erythrocytes ex vivo. Vet Res.

[22] Haatveit HM, Nyman IB, Markussen T, Wessel Ø, Dahle MK, Rimstad E. The non-structural protein μNS of piscine orthoreovirus 1 (PRV) forms viral factory-like structures. Vet Res, (in press) 10.1186/s13567-015-0302-0 PMC4705589 26743679

[23] Dahle MK, Wessel Ø, Timmerhaus G, NI B, Jørgensen SM, Rimstad E, Krasnov A (2015). Transcriptome analyses of Atlantic salmon (*Salmo salar L*.) erythrocytes infected with *Piscine orthoreovirus* (PRV). Fish Shellfish Immunol.

[24] Evensen Ø, Rimstad E (1990). Immunohistochemical identification of infectious pancreatic necrosis virus in paraffin-embedded tissues of Atlantic salmon (*Salmo salar*). J Vet Diagn Invest.

[25] Løvoll M, Wiik-Nielsen J, Grove S, Wiik-Nielsen CR, Kristoffersen AB, Faller R, Poppe T, Jung J, Pedamallu CS, Nederbragt AJ, Meyerson M, Rimstad E, Tengs T (2010). A novel totivirus and piscine reovirus (PRV) in Atlantic salmon (*Salmo salar*) with cardiomyopathy syndrome (CMS). Virol J.

[26] Løvoll M, Austbø L, Jorgensen JB, Rimstad E, Frost P (2011). Transcription of reference genes used for quantitative RT-PCR in Atlantic salmon is affected by viral infection. Vet Res.

[27] Hodneland K, Endresen C (2006). Sensitive and specific detection of Salmonid alphavirus using real-time PCR (TaqMan). J Virol Methods.

[28] Ørpetveit I, Mikalsen AB, Sindre H, Evensen Ø, Dannevig BH, Midtlyng PJ (2010). Detection of infectious pancreatic necrosis virus in subclinically infected Atlantic salmon by virus isolation in cell culture or real-time reverse transcription polymerase chain reaction: influence of sample preservation and storage. J Vet Diagn Invest.

[29] Chico V, Gomez N, Estepa A, Perez L (2006). Rapid detection and quantitation of viral hemorrhagic septicemia virus in experimentally challenged rainbow trout by real-time RT-PCR. J Virol Methods.

[30] Markussen T, Jonassen CM, Numanovic S, Braaen S, Hjortaas M, Nilsen H, Mjaaland S (2008). Evolutionary mechanisms involved in the virulence of infectious salmon anaemia virus (ISAV), a piscine orthomyxovirus. Virology.

[31] Kipar A, Meli ML (2014). Feline infectious peritonitis: still an enigma?. Vet Pathol.

[32] Watters SA, Mlcochova P, Gupta RK (2013). Macrophages: the neglected barrier to eradication. Curr Opin Infect Dis.

[33] Revie D, Salahuddin SZ (2014). Role of macrophages and monocytes in hepatitis C virus infections. World J Gastroenterol.

[34] Pantin-Jackwood MJ, Spackman E, Day JM (2007). Pathology and virus tissue distribution of Turkey origin reoviruses in experimentally infected Turkey poults. Vet Pathol.

[35] O'Hara D, Patrick M, Cepica D, Coombs KM, Duncan R (2001). Avian reovirus major mu-class outer capsid protein influences efficiency of productive macrophage infection in a virus strain-specific manner. J Virol.

[36] Tang H (2009) Regulation and function of the melanization reaction in Drosophila. Fly 3:105–111 10.4161/fly.3.1.7747 19164947

